# Synthesis and characterization of [Ce(BTC)]-MOF based capacitive and impedimetric dual-mode relative humidity sensor

**DOI:** 10.1039/d5ra06933e

**Published:** 2025-12-08

**Authors:** Eisha Maryam, Saeeza Riaz, Rabia Raza, Muhammad Tariq Saeed Chani, Qayyum Zafar

**Affiliations:** a Department of Chemistry, Lahore College for Women University 54000 Lahore Pakistan rabiakhan_star@yahoo.com; b Center of Excellence for Advanced Materials Research, King Abdulaziz University P. O. Box 80203 Jeddah 21589 Saudi Arabia; c Department of Physics, University of Management and Technology 54000 Lahore Pakistan qayyumzafar@gmail.com

## Abstract

A dual-mode relative humidity (RH) sensor based on a cerium 1,3,5-benzenetricarboxylate metal–organic framework ([Ce(BTC)]-MOF) was synthesized and integrated into an Al/[Ce(BTC)]-MOF/Al surface-type device. The MOF was prepared *via* a mild solvothermal route and characterized by FTIR, X-ray diffraction and scanning electron microscopy, revealing a crystalline monoclinic phase with nanocrystallites (∼67 nm) and a highly porous, fibrous morphology favorable for water adsorption. The humidity sensor demonstrated both capacitive and impedimetric operation when tested between 40 and 87% RH at 1 kHz, 10 kHz and 100 kHz. At 1 kHz the capacitance increased by over three orders of magnitude, giving a sensitivity of 2924 pF/% RH, while the impedance decreased nearly a thousand-fold with a impedimetric sensitivity of −40 kΩ/% RH. The device exhibited a response time of ∼125 s and a recovery time of ∼12 s in capacitive mode, demonstrating reliable and repeatable performance. These results confirm that the [Ce(BTC)]-MOF thin film provides an efficient dual-mode sensing platform that combines high sensitivity with simple fabrication, making it a promising candidate for next-generation humidity-monitoring applications.

## Abstract 2

The manuscript reports on the development of a dual-mode (capacitive and impedimetric) relative humidity sensor utilizing a highly crystalline and porous Ce(BTC)-MOF thin film.

## Graphical abstract

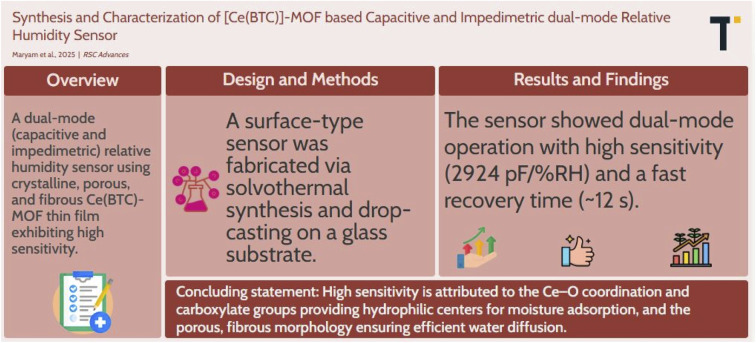

## 1. Introduction

Relative humidity (RH) is expressed as a percentage, which indicates a present state of absolute humidity relative to the saturation level at a given temperature. The accurate and reliable humidity measurement and the performance of humidity sensors for environmental monitoring are important issues in our daily life. Moreover, humidity sensors find extensive applications in various domains, including food preservation, agriculture, climatology.^1^ Humidity sensors exploiting impedimetric, capacitive, gravimetric, and optical signaling, based on different transduction mechanisms, have recently been engineered.^2,3^ However, impedimetric and capacitive humidity sensors have received increased attention and are consequently widely investigated.^4^ Impedimetric sensors typically depend on variations in electron transport phenomena between the sensing medium and the contact electrodes.^5^ On the other hand, capacitive sensors primarily monitor alterations in the effective dielectric characteristics of the sensing material in response to the variation in relative humidity.^6^ Capacitive humidity sensors present advantages such as ease of fabrication, minimal power consumption, and relatively better stability in response.^7,8^ Hence, it is crucial to innovate new capacitive humidity sensors that exhibit improved operational efficacy and incorporate novel sensing materials.

In the past decades, various materials have been utilized to fabricate capacitive and impedimetric humidity sensors. For instance, hydrophilic polymers offer the benefits of facile solution processing; however, they are compromised by poor stability in high-humidity environments. Quantum dots have also attracted excessive attention owing to their small nanoscale size and wide tunable bandgap.^9^ Similarly, humidity sensors based on metal oxides demonstrate superior sensing characteristics and thermal robustness, yet they require external heating and exhibit complex operational procedures.^10,11^ D. Wang *et al.*, previously reported poly(vinyl alcohol)/Ti_3_C_2_T_*x*_ (PVA/MXene) nanofibers film based piezoelectric nanogenerator (PENG) exhibiting fast response/recovery time of 0.9/6.3 s and stable repeatability.^12^ To enhance the overall sensitivity of humidity sensing devices, the most effective approach demands enhancing humidity adsorption performance by optimizing the surface area.^13^ Specifically, smaller-sized ZIF-8 (≈50 nm) offered much higher sensitivity for low-humidity capacitive sensing due to its larger surface area, but this came at the cost of increased fabrication complexity or reduced stability compared to larger particles. Consequently, there exists a necessity to investigate novel materials exhibiting strong hydrophilicity, high porosity, good stability and substantially greater surface areas compared with traditional sensor materials.^14,15^

This objective can be accomplished by synthesizing novel materials possessing accessible metal sites or hydrophilic functional groups. Recently, metal–organic frameworks (MOFs)—a specialized class of hybrid materials—have gathered substantial interest from numerous researchers across diverse fields, including catalysis, sensing, gas storage, and separation.^16–19^ Metal–organic frameworks (MOFs) are characterized by their customizable chemical functionalities, milder synthesis conditions and highly porous architecture, with surface areas capable of reaching values up to 7000 m^2^ g^−1^.^20,21^ Hence, MOFs in general, may serve as an ideal platform for humidity sensing due to their favorable water adsorption capabilities, uniform pore dimensions, high stability, and adaptable design potential compared to traditional amorphous materials, metal oxides, and polymers.^22^ However, despite their distinctive structural attributes and remarkable capacity for water adsorption, MOF materials have been infrequently employed in the production of real-time humidity sensing devices.^23^

B. Zhang *et al.*, have previously confirmed that the specific adsorption of water molecules may be observed at open metal sites, hydroxyls, or other polar (hydrophilic) surface groups which serve as the nucleation centers for water interaction.^24^ For instance, Zhang *et al.*, earlier synthesized NH_2_-MIL-125(Ti) MOF with desirable metal–oxygen groups (Ti–O) and amino groups (–NH_2_) acting as hydrophilic groups. The fabricated NH_2_-MIL-125(Ti) based humidity sensor showed a consistent decrease in the impedance with increase in humidity yielding a response of 27.5 and the response time and recovery time as 45 s and 50 s, respectively.^25^ Similarly, Yin *et al.* synthesized novel MOF [Cd(TMA)(DPP)_0.5_·H_2_O]_*n*_*via* facile precipitation method which exhibited O, N, and uncoordinated S atoms making its structure hydrophilic, and the resultant MOF-based sensor showed a promising response of ∼350 and response/recovery time ∼11 and 56 s, respectively.^26^ Recently, S. J. Kim *et al.*, demonstrated capacitive humidity sensing capability of ZIF-8, a stable MOF in low humidity levels and observed a maximum sensitivity of 5.34 pF/% RH under 500 Hz frequency.^13^

In the present study, we report a simplistic realization of surface type humidity sensor (Al/[Ce(BTC)]-MOF/Al) based on Ce-BTC MOF (BTC = 1,3,5-benzenetricarboxylic acid). The fabricated sensor has been operated at multiple frequencies of input bias and its electrical response (capacitance and impedance) has been inspected at wide-ranging ambient humidity levels. In this context, the [Ce(BTC)]-MOF offers an attractive platform because the Ce–O coordination and carboxylate functional groups provide abundant hydrophilic centers for moisture adsorption, while its porous and fibrous morphology ensures efficient diffusion of water molecules through the framework. These distinctive features are expected to promote enhanced dielectric polarization and ionic conduction, enabling high sensitivity and reproducibility. Moreover, the simple, low-temperature drop-casting approach adopted for device fabrication provides a cost-effective and scalable alternative to conventional high-temperature or lithography-based techniques. Collectively, these attributes form the motivation for exploring [Ce(BTC)]-MOF as a novel, dual-mode (capacitive and impedimetric) humidity sensing material with the potential for superior performance and facile integration into practical sensor platforms.

## 2. Experimental

### 2.1. Synthesis of [Ce(BTC)]-MOF

**Fig. 1:**
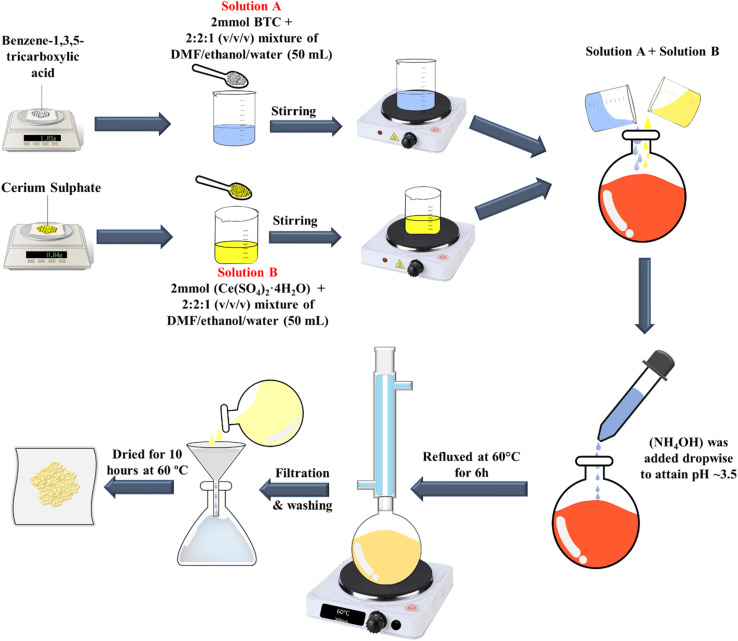
Pictorial representation of synthesis procedure of the synthesized [Ce(BTC)]-MOF.

The [Ce(BTC)]-MOF was prepared using the following protocol: primarily, solution A was formed by mixing 2 mmol of 1,3,5-benzene tricarboxylic acid (BTC) in 2 : 2 : 1 (v/v/v) mixture of DMF/ethanol/water (50 mL). After nearly 30 minutes of stirring at room temperature, the BTC-ligand was dissolved well and a clear solution was obtained. Solution B was prepared, by mixing 2 mmol of cerium(iv) sulfate tetrahydrate (Ce(SO_4_)_2_·4H_2_O) in 2 : 2 : 1 (v/v/v) mixture of DMF/ethanol/water (50 mL). The two solutions were then combined. To adjust the pH value, ammonium hydroxide (NH_4_OH) was introduced dropwise, until the pH reached approximately 3.5. The resulting mixture underwent reflux at 60 °C for a duration of 6 hours. Subsequently, it was allowed to cool to room temperature over a period of 6 hours. The yellowish-white crystals were acquired and separated by filtration from mother liquor. The resultant product was washed with DMF, ethanol, and water to remove possible impurities. Finally, crystals were dried for 10 hours at 60 °C before further analysis. Fig. 1, illustrates the synthesis route adopted for the synthesized specimen of [Ce(BTC)]-MOF, respectively.

### 2.2. Fabrication of humidity sensor

The humidity sensor was produced in a surface-type layout on a glass substrate using [Ce(BTC)]-MOF as the active sensing layer. Common soda lime glass slides (dimensions ∼25 × 25 × 1 mm) were selected to serve as the substrate for constructing the device. The glass slides underwent a two-step cleaning process: first gently rubbed with a lint-free wipe and a cotton swab soaked in soapy water. Subsequently, a conventional cleaning method was employed utilizing an Elmasonic E 30H ultrasonic cleaner. Specifically, the slides were subjected to ultrasonic cleaning for 15 minutes each with acetone, ethanol, and DI water, followed by drying in a dust-free setting with a dry air stream. Employing a shadow mask technique, an aluminium thin film with an average thickness of 150 nm was applied to the glass substrate *via* a customized physical vapor deposition (PVD) system at a deposition rate of 0.3 nm s^−1^. The deposition rate was monitored by *in situ* quartz crystal microbalance (QCM) integrated with the thermal evaporation system, whereas, the thickness of the thin film was determined using a surface profilometer (PLA-Tencor-P7).

**Fig. 2:**
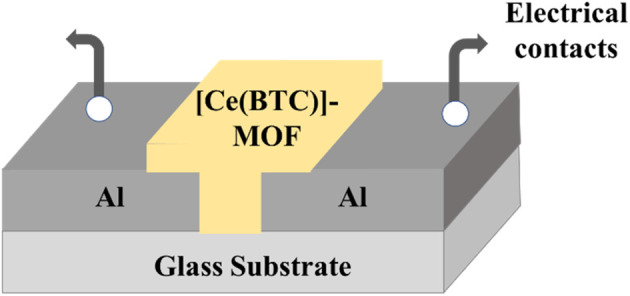
Schematic 3-D interpretation of the [Ce(BTC)]-MOF based humidity sensor.

The PVD system features a single-stage rotary vane pump (Pfeiffer, Hena 25, pumping speed ∼25 m^3^ h^−1^) and a diffusion pump (Agilent Technologies, VHS-4, pumping speed ∼750 L s^−1^), both utilized to evacuate the system chamber to 6 × 10^−4^ mbar (0.06 Pa). A shadow mask was employed to define a spacing of ∼40 µm between the pair of aluminium contact pads, to facilitate electrical connections to the humidity sensor. For the deposition of the [Ce(BTC)]-MOF active sensing layer, a 20 mg per mL [Ce(BTC)]-MOF solution was prepared and stirred overnight using magnetic stirring. The solution was subsequently filtered by passing it through Polytetrafluoroethylene (PTFE) membrane filters with a pore size of 0.45 µm. A 150 µL solution of [Ce(BTC)]-MOF in Dimethylformamide (DMF) was dropcasted to create a dielectric thin film covering the gap between the aluminium electrical contact pads. This process resulted in the observation of an average thickness of ∼180 nm for the [Ce(BTC)]-MOF thin film sensing layer as measured by the surface profilometer (PLA-Tencor-P7). A cross-sectional schematic representation of the Al/[Ce(BTC)]-MOF/Al planar humidity sensor is depicted in Fig. 2.

### 2.3. Sensor testing methodology

#### 2.3.1. Physical characterization

The UV-visible spectrum of the humidity sensing-material (in solution) was recorded using the Jasco V-770 spectrophotometer. The Nova NanoSEM 450 Field-Emission Scanning Electron Microscope (FE-SEM) was employed to examine of the surface morphology of the active sensing layer. The structural characteristics of the [Ce(BTC)]-MOF active layer have been explored through the analysis of the X-ray Diffraction (XRD) pattern utilizing the Shimadzu 7000 diffractometer, operating with Cu Kα1 radiation (*λ* = 0.15406 nm) produced at 30 kV and 30 mA, with a scan rate of 2° min^−1^ for 2*θ* values ranging from 5° to 60°.

#### 2.3.2. Electrical characterization

The laboratory-assembled chamber used for fabricated sensor testing was hermetically sealed. Control of humidity levels within the chamber was achieved through the regulation of dry and humid air flow, which passed through inlet and outlet valves. The humid air was generated by a commercially available humidifier while the dry air stream was used to decrease % RH level. The relative flows of the two streams were adjusted to achieve the desired percentage relative humidity (RH) setpoints. In our setup the typical reproducibility of the controlled RH was ±0.5% RH and the setpoint was considered stable when the reference hygrometer reading varied by less than ±0.2 % RH for 30 s. Monitoring of reference levels (such as relative humidity and ambient temperature) in the controlled environmental chamber was effectively carried out using the Pro's Kit MT 4014 commercial Thermo-hygrometer, offering a resolution of approximately 0.1% RH and 0.1 °C.

**Fig. 3:**
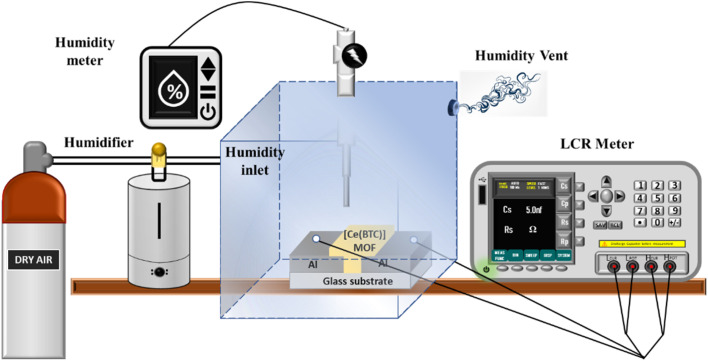
The general characterization setup used for testing of Al/[Ce(BTC)]-MOF/Al humidity sensor.

The characterization of the proposed surface type Al/[Ce(BTC)]-MOF/Al sensor focused on its electrical properties, achieved by subjecting it to various humidity levels with a measurement accuracy of 0.1% using the APPLENT AT2816B LCR Meter. Additionally, the sensor's electrical response was measured at three specific frequencies of the input signal (1 kHz, 10 kHz, and 100 kHz), while maintaining a constant applied bias (*V*_rms_) of 1.0 V. The schematic representation of the testing setup designed for the calibration of humidity sensors can be observed in Fig. 3.

## 3. Results and discussion

### 3.1. Physical characterization of [Ce(BTC)]-MOF

#### 3.1.1. Optical study of [Ce(BTC)]-MOF

**Fig. 4:**
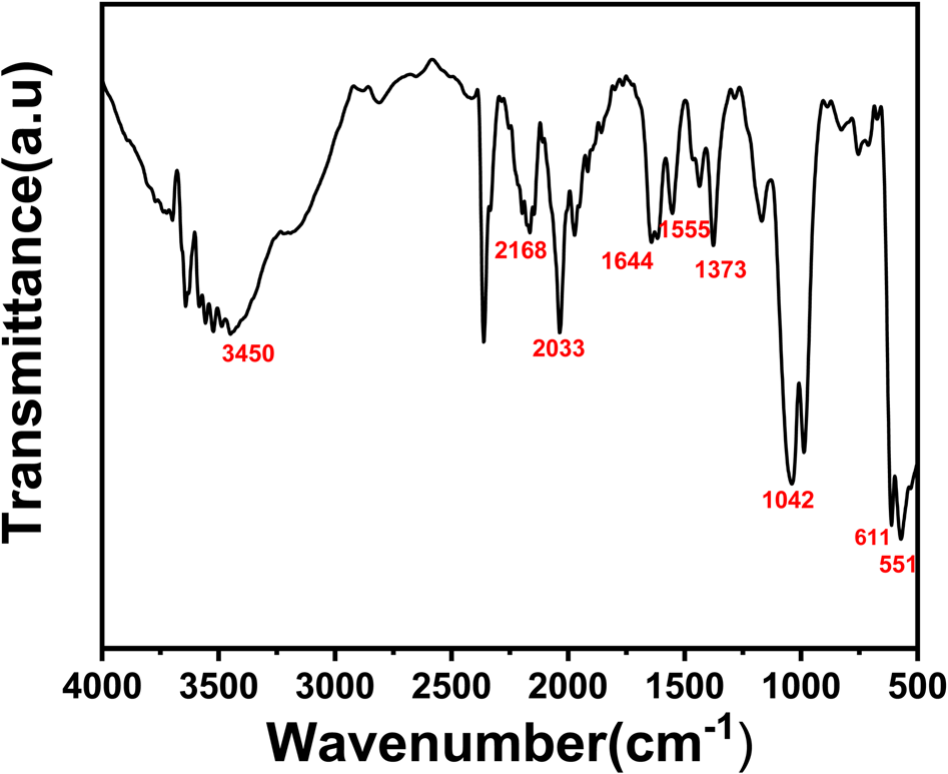
FTIR transmission spectrum of [Ce(BTC)]-MOF in powder form at room temperature.

**Fig. 5:**
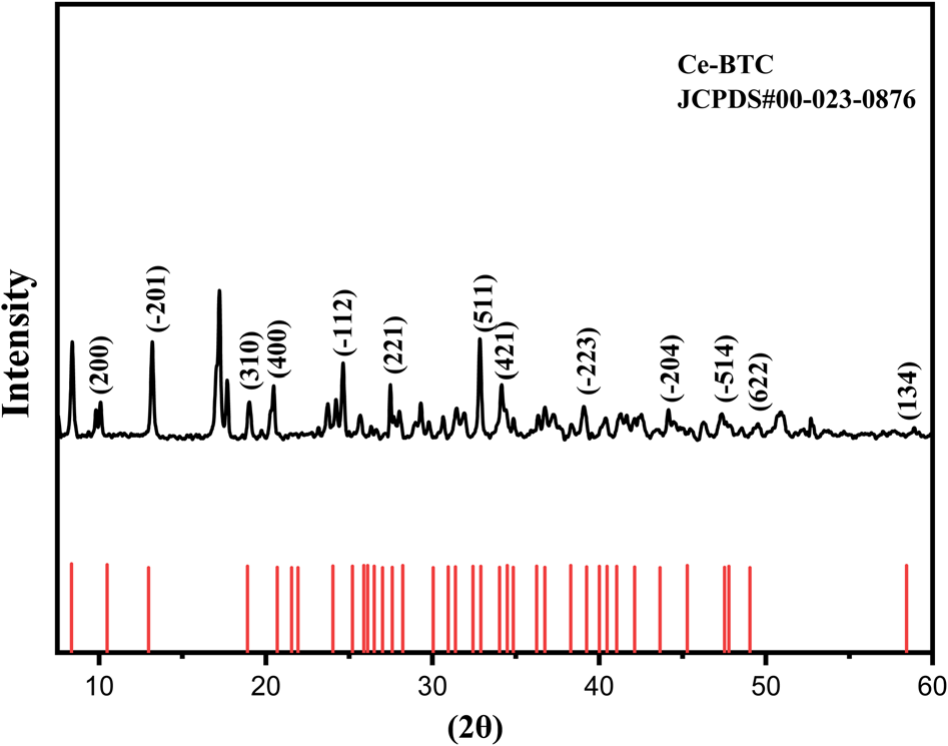
XRD diffractogram of [Ce(BTC)]-MOF thin film.

The FTIR spectra of the synthesized [Ce(BTC)]-MOF is illustrated in Fig. 4. It is evident that the spectrum of the synthesized product exhibits the distinctive bands at 1644 cm^−1^, associated with the asymmetric vibration (*ν*_asy_), and similarly at 1373 cm^−1^ which may be attributed to the symmetric vibrations (*ν*_sy_) of the COO^−^ group. The absorption peak detected at the wavelengths of 1555 cm^−1^ is indicative of the vibrational modes associated with the C=C bonds within the aromatic ring of the BTC^3−^ ligand. Additionally few minor peaks are also evident in Fig. 5, within the range of 500 and 700 cm^−1^ (specifically, at 611, and 551 cm^−1^), corresponding to Ce–O stretching vibrations of the synthesized [Ce(BTC)]-MOF product. The presence of these peaks proves that the Ce^3+^ ions have been successfully coordinated with the 1,3,5-H_3_BTC ligands. The broader peak detected at 3400–3300 cm^−1^ may be attributed to the stretching vibrations of hydroxyl (OH^−^) functional groups, signifying the presence of physically adsorbed or bonded (coordinated) water molecules on the [Ce(BTC)]-MOF sample surface.

The FTIR results indicate the presence of hydrophilic functional groups such as hydroxyl (–OH), carboxylate (–COO^−^), and Ce–O coordination sites on the [Ce(BTC)]-MOF framework. These polar surface groups are expected to serve as active adsorption centers for water molecules through hydrogen bonding and dipole–dipole interactions.^27,28^ During sensing, the initial chemisorption of water occurs at these hydrophilic sites, which subsequently promotes multilayer physisorption at higher % RH levels. This sequential adsorption mechanism enhances dielectric polarization and ionic conduction, thereby improving the overall sensitivity and response of the [Ce(BTC)]-MOF-based humidity sensor.

#### 3.1.2. Structural study of [Ce(BTC)]-MOF

Thin film X-ray diffraction (XRD) pattern of [Ce(BTC)]-MOF was obtained (see Fig. 5) to investigate the crystalline structure of the synthesized material. The observed diffraction peaks correspond well with the standard JCPDS card #00-023-0876, confirming the crystalline nature of the [Ce(BTC)]-MOF material. The synthesized MOFs exhibits a monoclinic crystal system that shows several distinct crystalline peaks at 2*θ* such as 8.40°,10.31°, 13.01°, 18.9°, 24.6°, 27.4°, 32.9°, 34.1°, 39.1°, 44.2°, 47.3°, 49.5°, 50.8°, and 58.8°, respectively. The presence of sharp and intense peaks, especially the ones indexed to (200) and (310), suggests well-defined crystallinity. In general, the alignment of the experimental XRD pattern with the theoretical Joint Committee on Powder Diffraction Standards (JCPDS) reference standard confirms the phase purity and structural integrity of the synthesized material, thereby verifying the successful synthesis of the target compound with no significant impurities. The average crystallite size of the synthesized material was estimated to be 67 nm from the XRD pattern using the Debye–Scherrer equation, which correlates the broadening of diffraction peaks with crystallite dimensions.

Note that while the JCPDS card #00-023-0876 primarily details reflections above 2*θ* ≈ 10°, the unindexed reflections observed at 2*θ* ≈ 8.4° and 17.3°, are consistent with previously reported Ce-BTC and related lanthanide-BTC structures.^29,30^ These low-angle peaks are typically associated with the long-range periodicity of the MOF framework, possibly arising from framework breathing or residual guest-molecule ordering and are considered intrinsic structural features rather than indications of impurity phases.

In addition, the well-defined crystalline monoclinic phase of the [Ce(BTC)]-MOF, as confirmed by XRD, suggests a stable framework with uniformly distributed Ce–O coordination sites. Such structural features are expected to facilitate the adsorption of polar water molecules and promote efficient charge transport during humidity sensing. Therefore, the presence of accessible metal–oxygen sites and an ordered crystal framework is anticipated to enhance the overall sensing response of the material.

#### 3.1.3 .Surface morphology of [Ce(BTC)]-MOF thin film

**Fig. 6:**
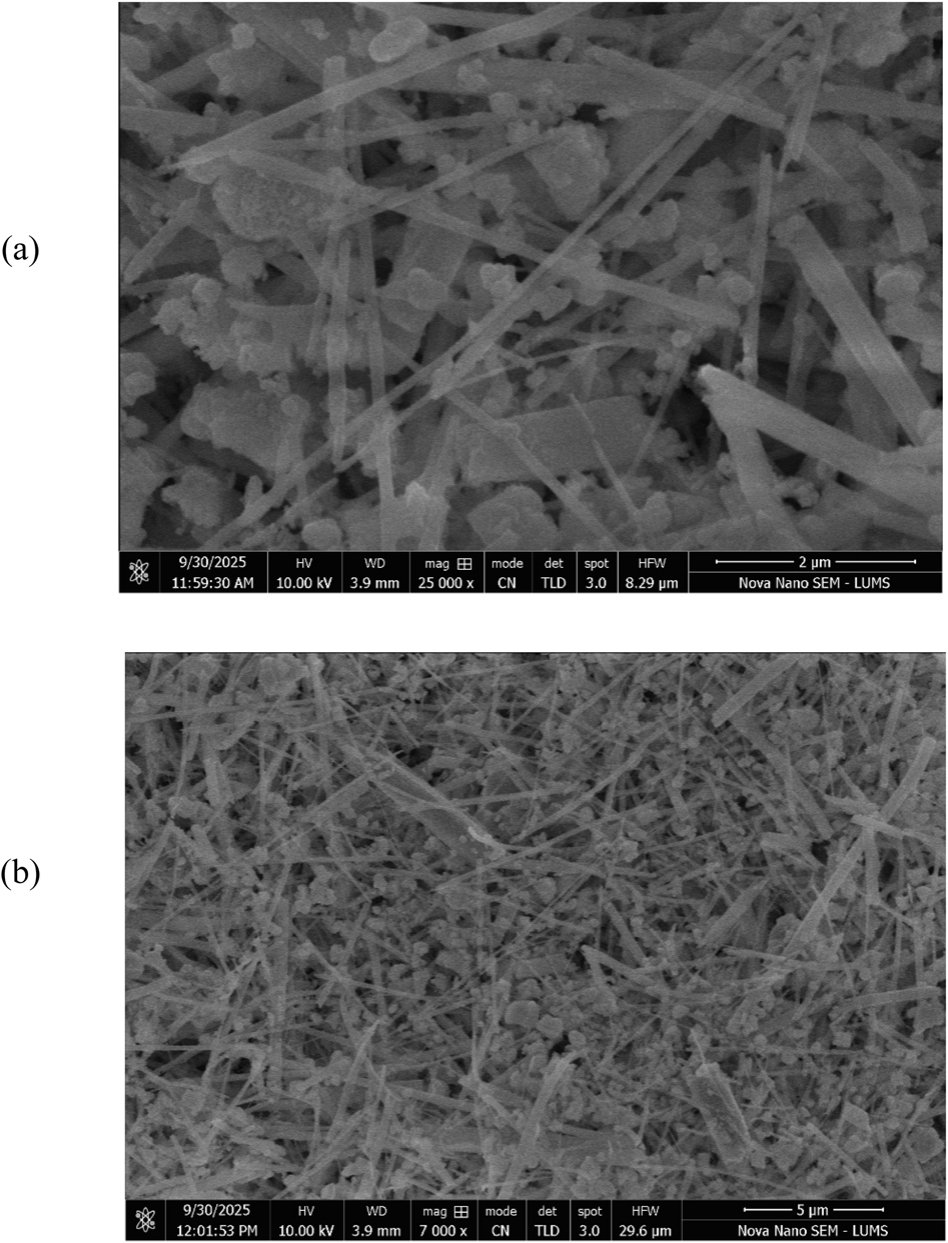
FESEM micrographs of [Ce(BTC)]-MOF active humidity sensing layer at (a) 25k and (b) 7k magnification scales.

The SEM images labeled as Fig. 6(a) and (b) display the surface morphology of a thin film composed of [Ce(BTC)]-MOF, intended for use as a humidity sensing layer. Both micrographs reveal a porous and loosely packed fibrous network, which is advantageous for humidity sensing due to the increased surface area and open channels that facilitate water molecule adsorption. The interconnected pores and open framework provide numerous active sites for water molecule adsorption, while the fibrous texture facilitates rapid capillary diffusion of moisture through the sensing layer. These structural features are anticipated to enhance the polarization and proton conduction pathways within the film, leading to a stronger capacitive and impedimetric response under varying humidity conditions. Hence, the distinctive morphology of [Ce(BTC)]-MOF thin film possesses sufficient active sites for water vapor interaction, making it a promising candidate for high-sensitivity humidity sensors.

### 3.2. Electrical characterization of humidity sensor

#### 3.2.1 .Humidity sensing performance study of [Ce(BTC)]-MOF

Humidity exerts a significant impact on a diverse array of physical, chemical, and biological phenomena, leading to potential applications in assessing fluctuations across various humidity levels. When utilized in capacitive configuration, the produced humidity sensor incorporates the [Ce(BTC)]-MOF sensing layer as a dielectric medium. The sensing material undergoes adsorption and desorption of moisture molecules in correlation with the ambient relative humidity while functioning in its capacitive operational mode. The dimensions of the aluminium electrodes (*A*), inter-electrodes separation (*d*), and the dielectric permittivity constant (*ε*_r_) of the [Ce(BTC)]-MOF dielectric substance affect the capacitance of the created apparatus (expressed quantitatively in eqn (1)).1
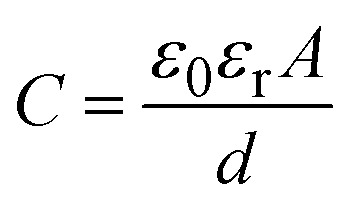
where “*C*” is the capacitance of the humidity sensor and “*ε*_0_” represents the dielectric permittivity of free space, “*A*” and “*d*” represent area of the electrodes and separation between them, respectively.

The dielectric permittivity of the active humidity sensing layer is influenced by the polarization occurring in the [Ce(BTC)]-MOF layer, whether it is humid or desiccated. There are typically four mechanisms, including dipolar, ionic, space charge, or electronic, that could potentially contribute to the polarizability of the active layer. The relationship between the dielectric constant (*ε*_r_), number density of molecules (*N*) and polarizability (*α*) is defined by the Clausius–Mosotti equation, as presented in eqn (2).2
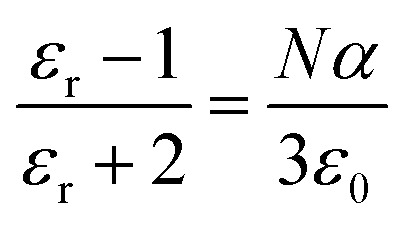
whereas eqn (3) describes the relationship between dielectric constant and capacitance.3
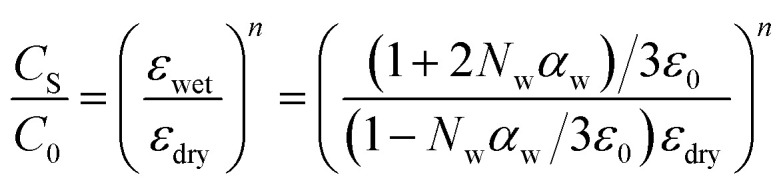
Here, *ε*_dry_ and *ε*_wet_ represent the relative dielectric constants of the dry and wet active sensing layer, respectively, while “*n*” denotes the dielectric morphology related factor. Typically, the dielectric permittivity of the desiccated organic semiconductor layer is approximately 5, which is notably lower compared to that of water, around 80. Consequently, as the [Ce(BTC)]-MOF thin layer continues to adsorb water molecules, the dielectric permittivity of the humid sensing layer undergoes significant variations.

**Fig. 7:**
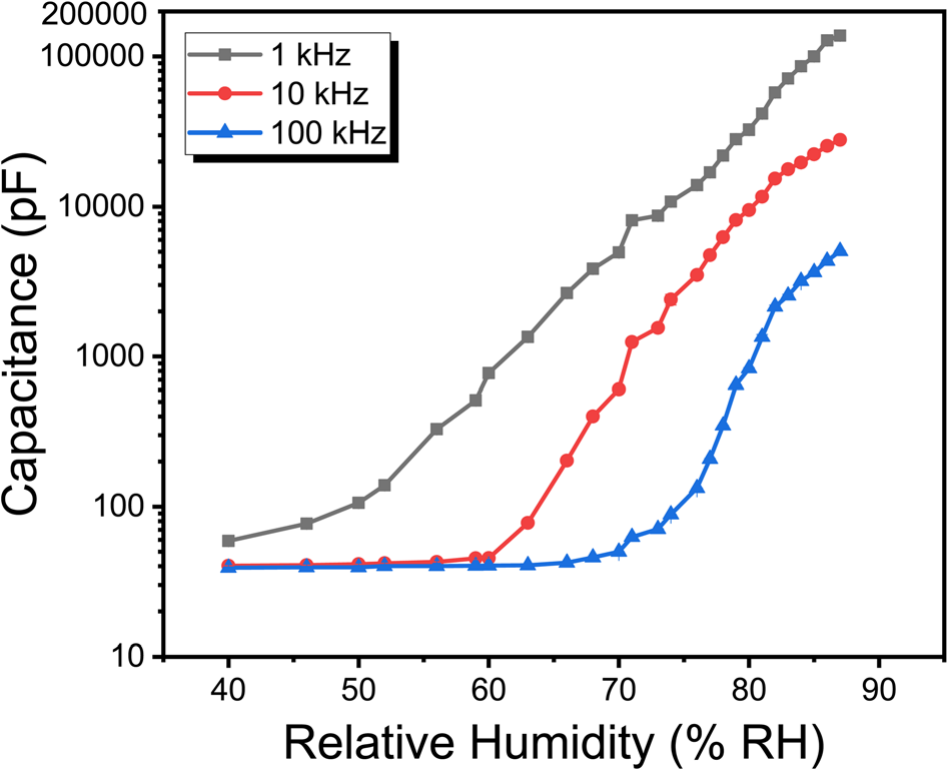
The impact of test frequencies on the capacitance *vs.* % RH response curves of [Ce(BTC)]-MOF based humidity sensor. (note: corresponding standard deviations (typically ±2–3%) are included as error bars in the plots, though they are not visually discernible due to their small size).

Fig. 7 illustrates the relationship between capacitance and relative humidity exhibited by the fabricated humidity sensor, covering a bandwidth of 40 to 87 % RH and tested at frequencies of 1 kHz, 10 kHz, and 100 kHz. Across all frequencies, the capacitance of the sensor rises quasilinearly in response to increasing % RH. Notably, at lower frequencies such as 1 kHz, variations in % RH have a more pronounced impact on capacitance compared to higher frequencies (10 kHz, and 100 kHz). Specifically, at a test frequency of approximately 1 kHz, the capacitance of the sensor amplifies by 2330.5 times when % RH increases from 40 to 87%. Similarly, the increase in capacitance at higher frequencies *i.e.*, 10 kHz, and 100 kHz has been observed to increase by a factor of 19.58, and 15.77 times, respectively. The sensor's sensitivity to ambient humidity at the three different AC test signal frequencies is measured to be 2924.27, 593.82 and 106.61 pF/% RH. It may be observed that the efficacy of the polarization mechanism decreases at higher frequencies, leading to a decline in the dielectric permittivity of the active layer.

**Fig. 8:**
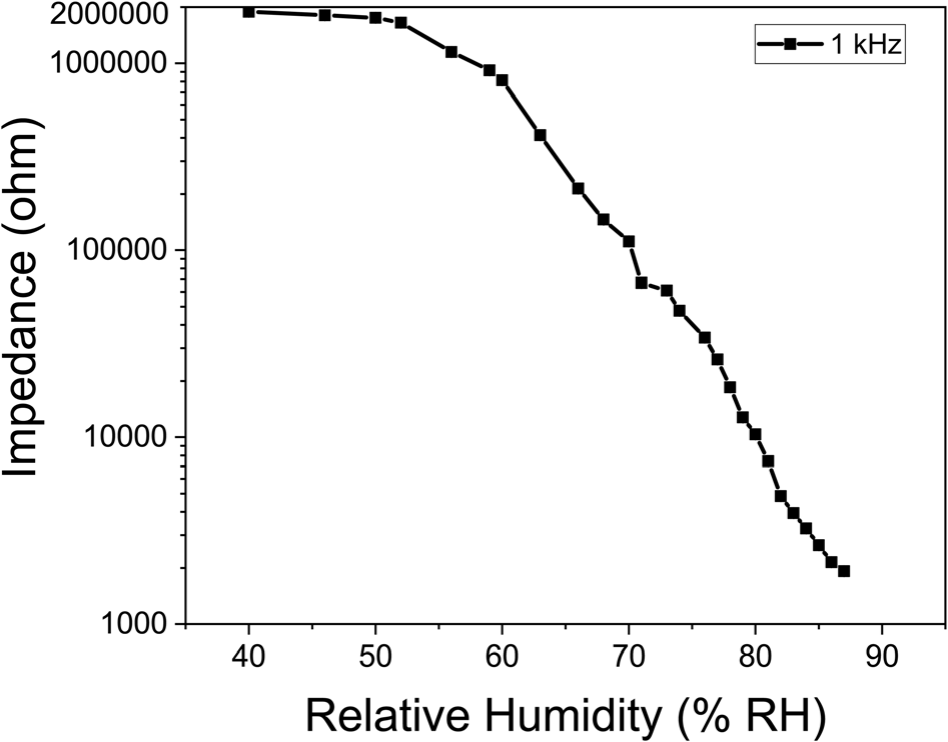
The impact of test frequencies on impedance *vs.* % RH response curves of [Ce(BTC)]-MOF based humidity sensor. (note: corresponding standard deviations (typically ±2–3%) are included as error bars in the plots, though they are not visually discernible due to their small size).

The variation in capacitance within the 40–55% RH range appears insignificant, as depicted in Fig. 8. It is well-established notion that the presence of water molecules on the active sensing layer is minimal under low ambient humidity conditions. Specifically, in the initial stages, the attachment of water molecules occurs through chemisorption (forming a monolayer) on the sensitive thin film due to surface electron deficiencies. Subsequently, multiple layers of physisorbed water molecules accumulate on the chemisorbed water layer as humidity levels increase. The introduction of additional adsorbed water molecules enhances the polarization effect, leading to a notable increase in sensor capacitance. Consequently, a consistent rise in capacitance is readily observed within the 55–87% RH range.

Furthermore, the repeatability and stability of the sensor's electrical capacitance were evaluated five times, yielding an estimated variation within the ±3.1% range. The sensor was also stored in an ambient environment for a duration of three months, during which an approximate 4.2% reduction in the capacitive response of the sensor was recorded at the operational frequency of 1 kHz.

The Metal organic Frameworks (MOFs) exhibit an intriguing charge transport property that is significantly influenced by ambient conditions, especially relative humidity. The impact of ambient relative humidity, ranging from 40% to 87% RH, on the impedance of the fabricated humidity sensor was observed at a 1 kHz test frequency, and illustrated in Fig. 8. It is evident that the sensor's impedance follows a consistent pattern, showing a decrease in magnitude as the ambient relative humidity increases. Specifically, at 1 kHz test frequency, there was a 984.37-fold change in electrical impedance between 40% RH and 87% RH, resulting in a sensitivity of −40.17 kΩ/% RH. This finding further demonstrates the efficacy of the [Ce(BTC)]-MOF semiconductor-based humidity sensor operating effectively in both capacitive and impedimetric modes for monitoring ambient relative humidity.

**Fig. 9:**
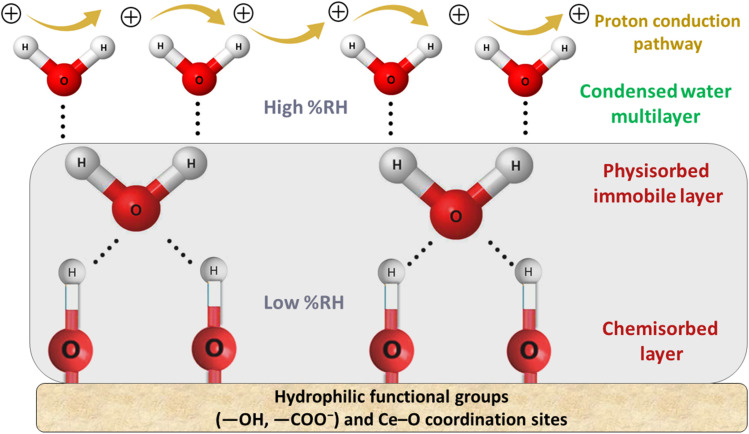
A schematic illustration depicting the mechanism of humidity sensor based on Grothus mechanism.

The operational mechanism of impedance-based sensors can be explained through the Grothus mechanism. Within the lower percentage relative humidity (% RH), an immobile layer of chemisorbed water molecules primarily forms on the [Ce(BTC)]-MOF thin film surface, with the conduction of the active layer at this phase being predominantly facilitated by intrinsic electrons, exclusively. As the % RH level increases, a multitude physisorbed water molecules adhere to the active sensing layer. These physisorbed layers demonstrate characteristics akin to a liquid and promptly disintegrate into hydronium ions (H_3_O)^+^ acting as charge carriers, as delineated in chemical eqn (4). Thus, the conductivity of the thin semiconductor film at heightened % RH levels is now governed by ionic conduction. In a larger context, a hydronium ion transfers a proton (H^+^) to an adjacent water molecule, perpetuating a cascade of reactions as shown in Fig. 9. The efficient proton transfer between neighboring molecules in the physisorbed H_2_O layers significantly diminishes the electrical impedance of the [Ce(BTC)]-MOF sensing layer.

4 H_2_O +H_2_O ⇌ H_3_O^+^ + HO^−^

**Fig. 10:**
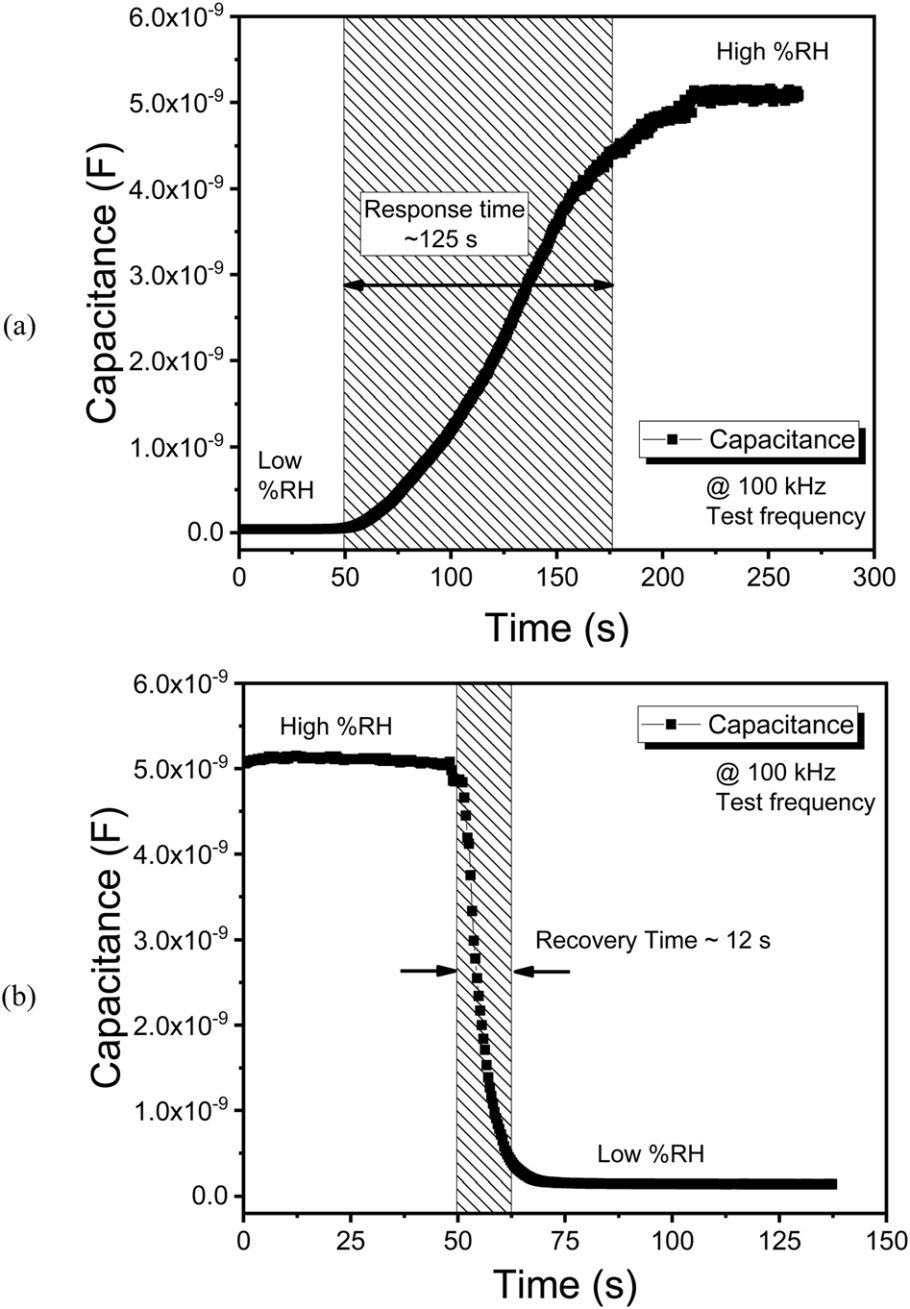
Estimation of the (a) response and (b) recovery time of the humidity sensor at 100 kHz test frequency.

When conducting an analysis of the sensor's performance, the response and recovery time, emerge as a pivotal parameter of considerable significance. This metric is calculated during the humidification/desiccation cycle of the dynamic curve associated with the humidity sensor.^31^ The temporal capacitive responses of the sensor to abrupt changes in ambient relative humidity levels are illustrated in Fig. 10(a) and (b). As illustrated in Fig. 10(a), the sensor operating in capacitive mode demonstrates a stable baseline at the initial measurement of low % RH; subsequently, with a step input in % RH transitioning from low to high the average response time has been assessed to be approximately 125 seconds. In a similar fashion, the reset time while functioning in capacitive mode has been documented at 12 seconds, as presented in Fig. 10(b). The notably fast response/recovery of the humidity sensor can be attributed to the effective diffusion of water molecules within the active sensing layer.

Table 1 provides a comparative analysis of the proposed [Ce(BTC)]-MOF based capacitive and impedimetric humidity sensor against previously reported sensors, focusing on essential performance metrics. While it exhibits a slight inefficiency regarding response/reset time, the proposed sensor demonstrates superior sensitivity in comparison to its counterparts. It is anticipated that through the careful selection of appropriate doping materials and their respective quantities, enhancements in sensitivity will occur alongside a significant reduction in response time. The effects of doping and geometrical parameters are currently being investigated and will be reported in future work.

**Table 1:** Comparison of humidity sensors based on key performance parameters

Material	Mode of operation	Sensitivity	Bandwidth	Response/reset time
DMBHPET^32^	Capacitive	0.007 pF/% RH	30–80% RH	10, 15 s
Polyimide^33^	Capacitive	22.29 pF/% RH	20–90% RH	25 s
Methyl-red^34^	Capacitive	16.92 pF/% RH	30–95% RH	∼10 s each
ZnO-SnO_2_ composite thin film^35^	Impedimetric	8.6 kΩ/% RH	32–92% RH	17, 65 s
Polyaniline/PVA^36^	Impedimetric	12.6 kΩ/% RH	30–85% RH	—
[Ce(BTC)]-MOF (current study)	Capacitive and impedimetric	146.17 pF/% RH 48.23 kΩ/% RH	39–85% RH	130, 156 s

## 4. Conclusion

A planar device utilizing the cerium 1,3,5-benzenetricarboxylate metal–organic framework [Ce(BTC)]-MOF was successfully developed and confirmed as a dual-mode relative humidity (RH) sensor. Structural characterization *via* XRD confirmed the formation of a highly crystalline MOF thin film with nanocrystallites (∼67 nm), while SEM confirmed its highly porous, fibrous morphology. FTIR analysis further confirmed the presence of hydrophilic functional groups (–OH, –COO^−^) and Ce–O coordination sites, which provide abundant active centers for efficient moisture adsorption *via* chemisorption and subsequent multilayer physisorption. The device demonstrated robust operation in both capacitive and impedimetric regimes. Electrical measurements showed a pronounced response to increasing RH, especially at lower frequencies (1 kHz). The capacitive mode exhibited an increase of over three orders of magnitude, achieving a high sensitivity of 2924 pF/% RH. Concurrently, the impedimetric mode showed a thousand-fold impedance decrease with a sensitivity of −40 kΩ/% RH while the response ∼125 s and recovery times ∼12 s are slightly longer than some polymer counterparts, the device outperforms many reported sensors in terms of sensitivity. These findings confirm that [Ce(BTC)]-MOF is a viable and versatile material, providing a strong foundation for future performance optimization through material tuning or device engineering for practical humidity-monitoring applications.

## Conflicts of interest

There are no conflicts to declare.

## Data Availability

The raw, original data from which the plots were generated is directly presented or accessible within the manuscript itself, in the format of data plots created using the software origin.
